# Effects of nitrogen availability on polymalic acid biosynthesis in the yeast-like fungus *Aureobasidium pullulans*

**DOI:** 10.1186/s12934-016-0547-y

**Published:** 2016-08-22

**Authors:** Yongkang Wang, Xiaodan Song, Yongjun Zhang, Bochu Wang, Xiang Zou

**Affiliations:** 1 College of Pharmaceutical Sciences, Chongqing Engineering Research Center for Pharmaceutical Process and Quality Control, Southwest University, 2 Tian Sheng Road, Beibei, Chongqing, 400715 People’s Republic of China; 2 Biotechnology Research Center, Southwest University, Chongqing, 400715 People’s Republic of China; 3 Ministry of Education, Key Laboratory of Biorheological Science and Technology (Chongqing University), Chongqing, 400044 People’s Republic of China

**Keywords:** *Aureobasidium pullulans*, Nitrogen availability, Polymalic acid, Proteomics, TOR signaling pathway, Transcriptomics

## Abstract

**Background:**

Polymalic acid (PMA) is a novel polyester polymer that has been broadly used in the medical and food industries. Its monomer, L-malic acid, is also a potential C4 platform chemical. However, little is known about the mechanism of PMA biosynthesis in the yeast-like fungus, *Aureobasidium pullulans*. In this study, the effects of different nitrogen concentration on cell growth and PMA biosynthesis were investigated via comparative transcriptomics and proteomics analyses, and a related signaling pathway was also evaluated.

**Results:**

A high final PMA titer of 44.00 ± 3.65 g/L (49.9 ± 4.14 g/L of malic acid after hydrolysis) was achieved in a 5-L fermentor under low nitrogen concentration (2 g/L of NH_4_NO_3_), which was 18.3 % higher yield than that obtained under high nitrogen concentration (10 g/L of NH_4_NO_3_). Comparative transcriptomics profiling revealed that a set of genes, related to the ribosome, ribosome biogenesis, proteasome, and nitrogen metabolism, were significantly up- or down-regulated under nitrogen sufficient conditions, which could be regulated by the TOR signaling pathway. Fourteen protein spots were identified via proteomics analysis, and were found to be associated with cell division and growth, energy metabolism, and the glycolytic pathway. qRT-PCR further confirmed that the expression levels of key genes involved in the PMA biosynthetic pathway (*GLK, CS, FUM, DAT*, and *MCL*) and the TOR signaling pathway (*GS*, *TOR1*, *Tap42,* and *Gat1*) were upregulated due to nitrogen limitation. Under rapamycin stress, PMA biosynthesis was obviously inhibited in a dose-dependent manner, and the transcription levels of *TOR1, MCL,* and *DAT* were also downregulated.

**Conclusions:**

The level of nitrogen could regulate cell growth and PMA biosynthesis. Low concentration of nitrogen was beneficial for PMA biosynthesis, which could upregulate the expression of key genes involved in the PMA biosynthesis pathway. Cell growth and PMA biosynthesis might be mediated by the TOR signaling pathway in response to nitrogen. This study will help us to deeply understand the molecular mechanisms of PMA biosynthesis, and to develop an effective process for the production of PMA and malic acid chemicals.

**Electronic supplementary material:**

The online version of this article (doi:10.1186/s12934-016-0547-y) contains supplementary material, which is available to authorized users.

## Background

Polymalic acid (PMA) is a novel polyester polymer composed of L-malic acid as the sole monomer [1, 2]. Due to its good biodegradability, water-solubility, and biocompatibility, it can be used as a new functional material for drug delivery, tissue scaffolds, and food materials, etc. [3, 4]. Its monomer, L-malic acid, which was widely used in the food and pharmaceutical industries, also does not demonstrate toxicity or immunogenicity in the body. In our previous study, a novel process was developed through acid hydrolysis of PMA for the production of L-malic acid [5]. In recent years, PMA and L-malic acid have attracted much attention due to their wide application.

Currently, PMA is mainly produced using the microorganism *Aureobasidium pullulans. A. pullulans* is a black yeast-like species that is popularly known as secreting melanin. *Aureobasidium* spp. are ubiquitous species that have evolved an extraordinary tolerance for a broad range of ecological conditions and can be isolated from pollen, leaves, and even antarctic soils, and deep sea water [6]. It is regarded as a polyextremotolerant organism that can survive in hypersaline, acidic and basic, cold and oligotrophic conditions. Due to its inherent universality, *A. pullulans* can produce a variety of metabolites, such as PMA, pullulan, melanin, amylase, proteinase, xylanase, liamocins, etc. [7, 8].

Culture stress is becoming an efficient strategy for the overproduction of various metabolites by microorganisms. Some genera of filamentous fungi, e.g., *Aspergillus* and *Rhizopus*, are known to produce large quantities of malic and fumaric acids and secrete them into the culture broth when cultured under stress conditions [9, 10]. Under nitrogen limitation, these fungi accumulate fumaric and malic acids as the end products of the reductive TCA cycle in the cytosol [11]. In addition, nitrogen limitation has become an important approach for regulating the biosynthesis of some biopolymers, such as polyhydroxyalkanoates (PHA) [12] and poly-3-hydroxybutyrate (PHB) [13]. In our previous study, ammonium nitrate (NH_4_NO_3_) was used as the most optimal nitrogen source for the production of PMA [5, 14, 15]; however, cell growth and PMA biosynthesis in response to nitrogen is not yet well understood.

In eukaryotic cells, most of the regulatory events that are induced by nitrogen availability are mediated through “TOR” (target of rapamycin) kinase and the downstream TOR signaling pathway. TOR is a conserved Ser/Thr kinase and plays a crucial role as a global regulator in cell growth and metabolism by sensing and integrating a variety of inputs arising from amino acids, cellular stresses, energy status, and growth factors [16–18]. In *Fusarium fujikuroi*, Teichert et al. firstly found that TOR kinase was involved in the nitrogen regulation of secondary metabolism. Inhibition of TOR by rapamycin could affect the expression of AreA-controlled secondary metabolite genes involved in the gibberellins (GAs) and bikaverin biosynthesis pathway [19]. Moreover, the TOR pathway was also confirmed to participate in regulating the virulence of *Fusarium graminearum* [20].

Although PMA has attracted much attention in different fields, the molecular basis of PMA biosynthesis is still unclear. In this study, the effects of different levels of NH_4_NO_3_ on cell growth and PMA biosynthesis were investigated on different scales. Comparative transcriptomics and proteomics analyses were used for a global understanding of the nitrogen response. Under rapamycin stress, the outputs of the TOR signaling pathway in *A. pullulan*s were to determine its role in regulating cell growth and PMA biosynthesis.

## Methods

### Cultures and media

The strain *A. pullulans* CCTCC M2012223 was isolated by our laboratory and can be obtained from the China Center for Type Culture Collection (Wuhan, China). This strain was maintained on the PDA slant. The seed culture medium contained 60, 2, 0.1, 0.1, 0.1, 0.5 and 20 g/L of glucose, NH_4_NO_3_, KH_2_PO_4_, MgSO_4_, ZnSO_4_, KCl and CaCO_3_, respectively. The seed culture was grown in a 500-mL shake flask containing 50 mL of liquid medium, and was incubated at 25 °C in a rotary shaker (180 rpm) for 2 days. The fermentation medium contained 90, 0.1, 0.1, 0.1, 0.5 and 30 g/L of glucose, KH_2_PO_4_, MgSO_4_, ZnSO_4_, KCl and CaCO_3_, respectively.

### Fermentation in shake flask and fermentor

In order to evaluate the effect of nitrogen concentrations on cell growth and PMA biosynthesis in different scales, the different levels of NH_4_NO_3_ from 0.1 to 10.0 g/L were taken in the initial fermentation medium, respectively. The shake flask fermentation was inoculated with 10 % (v/v) of the above-described seed culture medium and kept at 25 °C with shaking at 220 rpm for 4 day. Batch fermentation kinetics was studied in a 5-L stirred-tank fermentor (Shanghai Baoxing Co. Ltd, China) containing 3 L of the fermentation medium, as well as adding 0.1, 2 or 10 g/L of NH_4_NO_3_. The fermentation was inoculated with 300 mL of seed culture grown in a shake flask for 48 h, and operated at 25 °C with agitation and aeration at 400–600 rpm and 1.3 vvm, respectively. All trials were performed in triplicate.

### Transcriptomics analysis

As for transcriptomics and proteomics analyses, three independent cell samples under the condition of nitrogen limitation (2 g/L) and nitrogen repletion (10 g/L) were harvested from 5-L stirred-tank fermentor at 36 h, respectively. Total RNA was extracted using Fungal RNA Kit (Omega, USA), and the quality of extracted RNA was measured by NanoDrop 2000 (Thermo scientific, USA). After examination, the magnetic beads with Oligo (dT) are used to isolate mRNA. Mixed with the fragmentation buffer, the mRNA is fragmented into short fragments. Then cDNA is synthesized using the mRNA fragments as templates. Short fragments are purified and resolved with EB buffer for end reparation and single nucleotide A (adenine) addition. After that, short fragments are connected with adapters. The suitable fragments are selected for the PCR amplification as templates. At last, the library was sequenced using Illumina HiSeq™ 2500 equipment. The transcriptome data set of *A. pullulans* was deposited at NCBI Sequence Read Archive database (SRA—http://www.ncbi.nlm.nih.gov/Traces/sra/) with the BioProject ID PRJNA301913.

In a comparison analysis, two-class unpaired method in the significant analysis of microarray software (SAM, version 3.02) was performed to identify significantly differentially expressed genes between nitrogen limited and sufficient groups, determining with a selection threshold of false discovery rate, FDR <5 % and fold change ≥2. Raw data was log_2_-transformed and imported.

### Protein extraction and two-dimensional (2-D) electrophoresis

Total protein extracts from cells under nitrogen limited and sufficient conditions were prepared via the phenol extraction method. Proteins were separated by two-dimensional gel electrophoresis (2-DE), and the protein spots were visualized by silver staining. For each sample, at least three independent protein extracts and two 2-DE analyses were performed.

### 2-D gel electrophoresis analysis

In order to analyze the expressed protein patterns, the silver stained gels were scanned using a PowerLook 1100 scanner (UMAX, Taiwan), and protein spots images were analyzed using GE HealthCare software (Amersham Biosciences, Sweden). The protein spots with at least twofold differences in absolute abundance and reproducible changes were considered as the candidate proteins. These protein spots on the corresponding Coomassie-stained gels were excised and digested with trypsin using a Spot Handling Workstation (Amersham Biosciences, Sweden). The digested peptide masses were measured using a MALDI-TOF-TOF mass spectrometer (ABI 4700 system, USA). Data were processed via the Data Explorer software and proteins were identified by searching against a comprehensive non-redundant sequence database using the MASCOT search engine.

### Quantitative RT-PCR

The transcription levels of key genes involved in PMA biosynthetic pathway (e.g., *GLK, CS, FUM, DAT* and *MCL*) and TOR signaling pathway (e.g., *GS*, *TOR1*, *Tap42* and *Gat1*) were tested by quantitative RT-PCR. Total RNA samples from nitrogen limited or nitrogen sufficient fermentation at 36 h were extracted using Fungal RNA Kit (Omega, USA) and reversed to cDNA using reverse transcriptase (Takara, Japan). Primers of different genes were shown in Additional file 1: Table S1. The experiment was repeated three times. The quantitative PCR assay was performed according to Sybr Green method (qPCR Master Mix, ABI, USA) using fluorescence quantitative PCR (ABI, USA).

### Rapamycin sensitivity

In order to evaluate the effect of rapamycin on cell growth and PMA biosynthesis, *A. pullulans* cells were cultured on the plate and shake flask, respectively. In the plate culture, cells were spread on the PDA plate amended with rapamycin from 5 to 50 ng/mL. For the shake flask fermentation, the seed culture (10 %, v/v) was inoculated to 50 mL fermentation media containing various concentrations of rapamycin from 5 to 50 ng/mL. The fermentation cultivation was then operated at 25 °C with shaking at 180 rpm for 4 days. All trials were performed in triplicate. The cell samples at the plate with adding 10 ng/mL of rapamycin were extracted for quantitative PCR assay.

### Metabolites analysis

The cell density was determined by the cell dry weight (DCW) method. Prior to the measurement, excess CaCO_3_ was eliminated from the broth by adding 1 M HCl. The cell suspension was centrifuged at 4000×*g* and then dried overnight at 105 °C. The concentration of residual sugar was measured using the dinitrosalicylic acid assay method [21]. The concentration of residual NH_4_^+^ in the broth was detected by phenol-hypochlorite reaction [22]. For the analysis of intracellular ATP/ADP and NADH/NAD^+^ ratio, the methods have been introduced in our previous reports [14]. The experiment was repeated three times.

PMA was analyzed by centrifuging the fermentation broth and then adding 1 mL of resulted supernatant to 1 mL of 2 M H_2_SO_4_ in an incubator at 85 °C for 8 h. After neutralization of the solution, the hydrolyzed sample was analyzed by HPLC (Agilent 1200, USA), using a Spursil C18-EP organic acid column at 40 °C eluted with 5 mM H_2_SO_4_ at a rate of 0.6 mL/min to determine its malic acid content [23].

## Results

### Investigation of nitrogen availability on PMA biosynthesis

**Fig. 1:**
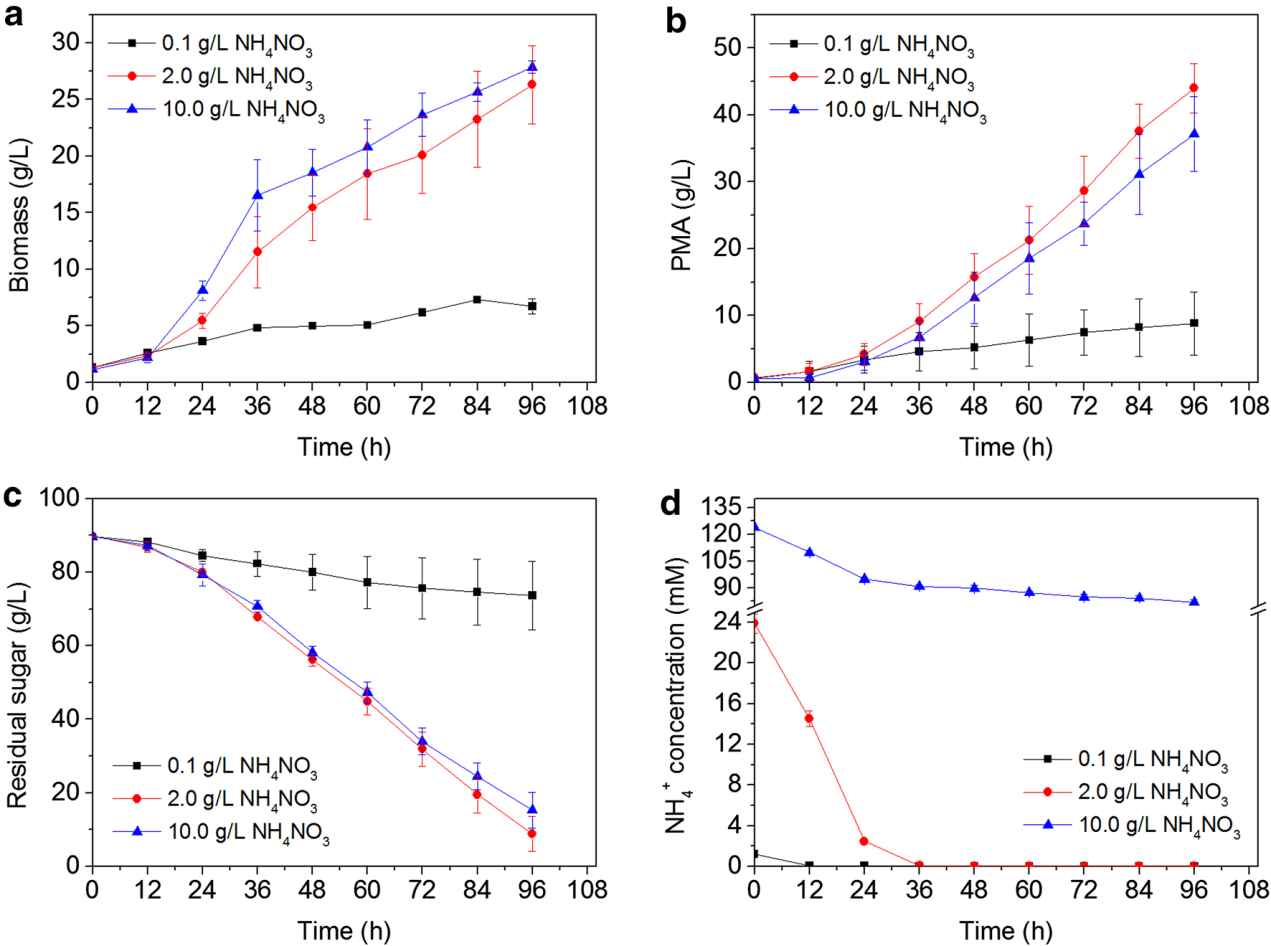
Time course of cell biomass (**a**), PMA production (**b**), residual sugar (**c**) and NH_4_^+^ concentration (**d**) under the different levels of NH_4_NO_3_ in a 5-L stirred-tank fermentor. Data are given as the average of triplicate experiments

In general, the utilization of a nitrogen source is essential to cell growth, however, the type of nitrogen source affects PMA biosynthesis [15]. In our previous study, NH_4_NO_3_ was selected as the best nitrogen source for the production of PMA [5]. Firstly, the effects of different NH_4_NO_3_ concentrations on cell growth and PMA biosynthesis were investigated in shake flasks. As shown in Table 1, for NH_4_NO_3_ concentrations within the range of 0.1 and 2 g/L, both cell growth and PMA production were gradually increased. The highest PMA titer reached 20.02 ± 2.81 at 2 g/L of NH_4_NO_3_, with a corresponding PMA yield (Yp/x) of 0. 96 g/g. However, when the NH_4_NO_3_ concentration was >2 g/L in the shake flasks, PMA biosynthesis seemed to be inhibited, as fermentation stopped and there was significant accumulation of residual glucose. At the high level of NH_4_NO_3_ (10 g/L), the PMA titer reached 16.57 ± 0.90 g/L, with a relatively low PMA yield (Yp/x) of 0. 69 g/g. It was concluded that the level of nitrogen (NH_4_NO_3_) in the media was an important factor to regulate PMA biosynthesis. Therefore, the kinetics of different levels of NH_4_NO_3_ (0.1, 2 and 10 g/L) were further investigated in a 5-L stirred-tank fermentor. It is evident from Fig. 1 that cell growth was associated with the level of nitrogen; the highest PMA titer of 44.0 ± 3.65 g/L (49.9 ± 4.14 g/L of malic acid after hydrolysis) was achieved at 2 g/L of NH_4_NO_3_ in 96 h, with a corresponding PMA productivity of 0.46 ± 0.04 g/L·h. At the lowest level of NH_4_NO_3_ (0.1 g/L), the NH_4_^+^ concentration was almost completely consumed at 12 h (only 0.05 mM), which did not support cell growth and seriously hindered PMA biosynthesis. However, at the highest level of NH_4_NO_3_ (10 g/L), although the cell growth was faster than that at the lower levels of NH_4_NO_3_ (0.1 or 2 g/L), the production of PMA was obviously decreased to 37.2 ± 4.58 g/L in 96 h, which might be attributed to the excess NH_4_^+^ concentration in the broth. It was noted that the NH_4_^+^ concentration was at a surplus until the end of fermentation (Fig. 1d). In comparison, at 2 g/L of NH_4_NO_3_, the NH_4_^+^ concentration was almost consumed up at 36 h (0.11 mM). These results indicated that high levels of nitrogen favor cell growth, while on the contrary, low levels of nitrogen are beneficial for PMA biosynthesis.

**Table 1:** Effects of different levels of NH_4_NO_3_ on cell growth and PMA biosynthesis in shake flasks

NH_4_NO_3_ (g/L)	Residual sugar (g/L)	Cell biomass (g/L)	PMA (g/L)	Productivity (g/L h)	Yield (Y_P/X_, g/g)	Yield (Y_P/S_, g/g)
0.1	56.67 ± 0.50	6.23 ± 0.71	6.17 ± 0.29	0.06 ± 0.003	1.00 ± 0.137	0.18 ± 0.011
0.5	40.77 ± 0.12	13.09 ± 1.09	10.86 ± 1.91	0.09 ± 0.042	0.85 ± 0.246	0.25 ± 0.081
1.0	24.57 ± 0.31	17.17 ± 0.77	14.01 ± 0.04	0.15 ± 0.000	0.82 ± 0.037	0.21 ± 0.000
2.0	16.95 ± 1.91	21.31 ± 3.81	20.02 ± 2.81	0.21 ± 0.029	0.96 ± 0.242	0.24 ± 0.032
4.0	16.70 ± 0.35	23.55 ± 0.85	17.31 ± 0.24	0.18 ± 0.003	0.74 ± 0.024	0.24 ± 0.002
10.0	14.52 ± 0.58	24.20 ± 1.96	16.57 ± 0.90	0.17 ± 0.009	0.69 ± 0.081	0.22 ± 0.013

All the values are the means and standard deviations of three independent experiments. Yield (Yp/x): the ratio of PMA to cell biomass concentration (g/g); Yield (Yp/s): the ratio of PMA to consumed sugar concentration (g/g)

**Fig. 2:**
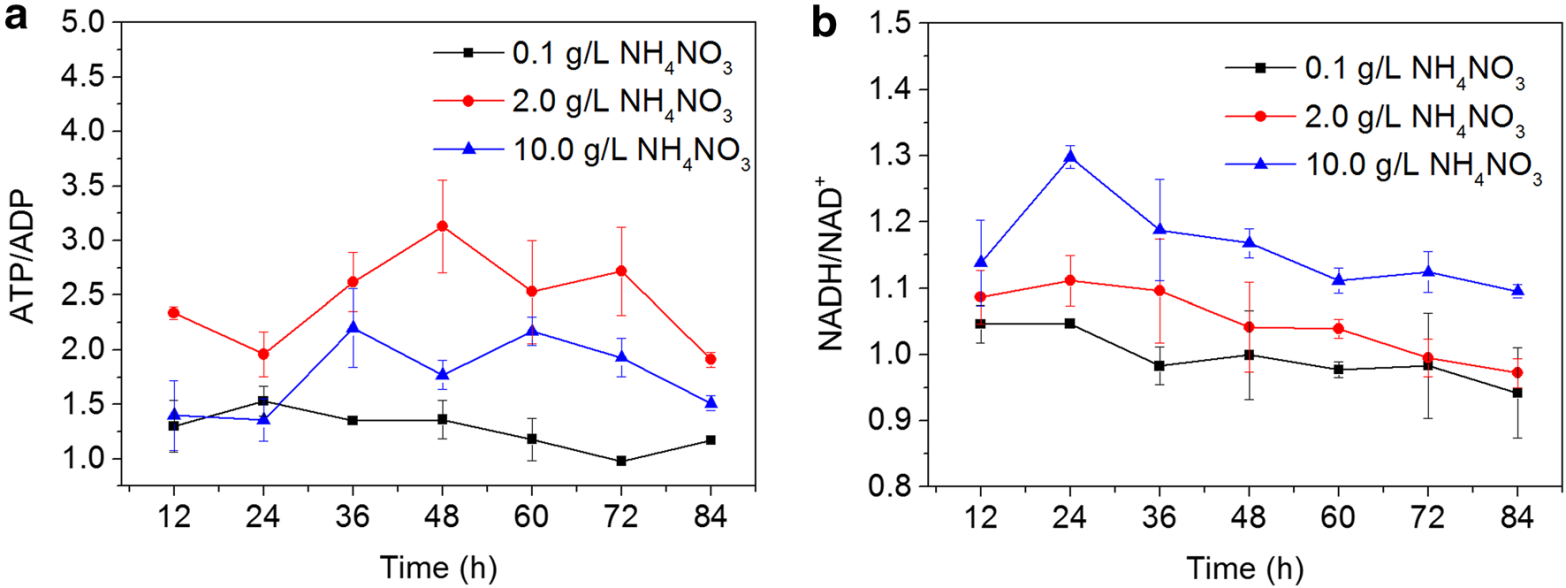
The ratios of ATP/ADP and NADH/NAD^+^ under the different levels of NH_4_NO_3_. **a** ATP/ADP; **b** NADH/NAD^+^. Data are given as the average of triplicate experiments

Figure 2 showed intracellular variation in the ratios of NADH/NAD^+^ and ATP/ADP under different levels of NH_4_NO_3_. Compared to 0.1 g/L of NH_4_NO_3_, the ratio of ATP/ADP and NADH/NAD^+^ at 2 and 10 g/L of NH_4_NO_3_ remained at relatively high levels, respectively, which might be helpful for high cell growth and metabolism. However, the ratio of NADH/NAD^+^ at 10 g/L of NH_4_NO_3_ was obviously higher than that at 2 g/L of NH_4_NO_3_ during the whole fermentation process, which should generate more ATP necessary for cell metabolism through oxidative phosphorylation. However, the ratio of ATP/ADP at 10 g/L of NH_4_NO_3_ was lower than that at 2 g/L of NH_4_NO_3_. These results also revealed that the level of nitrogen might be involved in regulating the cofactor regeneration and energy supply during the fermentation process.

### Transcriptomics and proteomics analysis

**Fig. 3:**
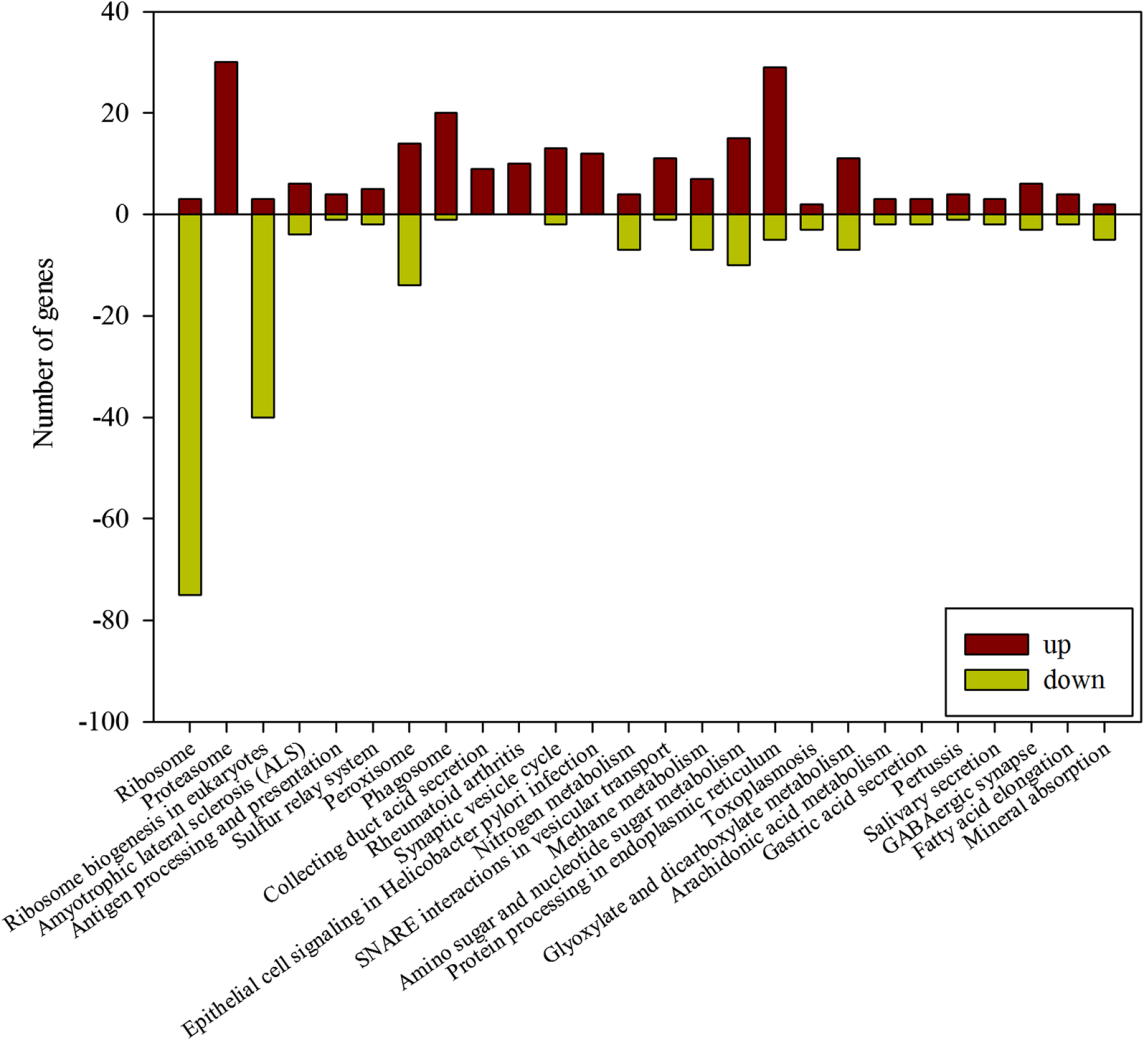
Major function categories of differentially expressed genes under nitrogen-sufficient conditions

In order to further understand cell growth and PMA biosynthesis in response to nitrogen levels, the global transcriptional profiles under nitrogen repletion (10 g/L of NH_4_NO_3_) and nitrogen limitation (2 g/L of NH_4_NO_3_) were examined at 36 h. In this time node, the NH_4_^+^ concentration under nitrogen limitation (2 g/L) was consumed completely, whereas it was sufficient under nitrogen repletion (10 g/L), as previously described. As shown in Additional file 2: Fig. S1, a total of 3706 differentially expressed genes (DEGs), including 1885 upregulated and 1821 downregulated genes, were identified under nitrogen sufficient condition. The enrichment analysis of the KEGG pathway showed that these differentially expressed genes were mainly focused on the ribosome, ribosome biogenesis, proteasome, and nitrogen metabolism (*p* < 0.05) (Additional file 3: Table S2; Fig. 3), which was associated with cell growth and proliferation that can be mediated by the TOR signaling pathway. Furthermore, these differentially expressed genes were categorized into three major functional groups, including those for biological processes, cellular components, and molecular function via gene ontology (GO) analysis (data no shown). In the biological process group, these significantly expressed genes were mostly involved in metabolic, cellular, and biological regulation processes. In the cellular component group, most of the differentially expressed genes were linked to macromolecular complexes, membranes and organelles, and their molecular functions were primarily focused on catalytic, binding, and transport activity. These results indicated that cell growth and metabolism could be significantly regulated by the level of nitrogen.

**Fig. 4:**
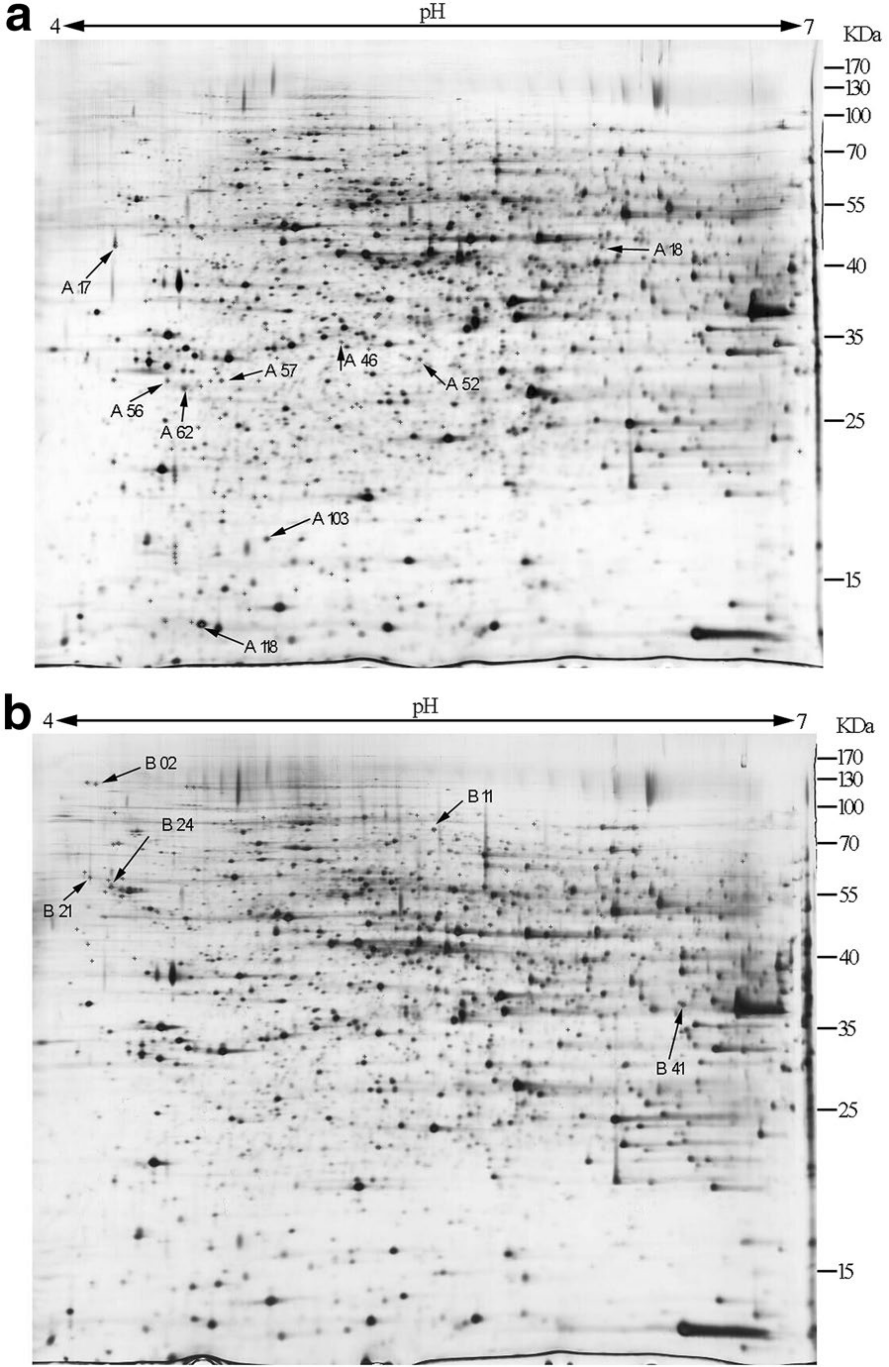
Comparative 2-DE gel analyses of cellular proteins of *A. pullulans* cultivated under nitrogen-sufficient (**a**) and nitrogen-limited (**b**) conditions. *Spots* marked on the maps with *arrows* and *numbers* represent the 14 identified proteins analyzed with MALDI-TOF/TOF

Figure 4 shows representational gel maps of the proteins extracted from *A. pullulans* cells at 36 h under nitrogen limitation (2 g/L of NH_4_NO_3_) and nitrogen repletion (10 g/L of NH_4_NO_3_). Based on the comparison and analysis of the gel images, a total of 27 protein spots from the whole cell protein gels were excised and subjected to MALDI-TOF/TOF analysis. Fourteen protein spots were successfully identified, and were matched to known proteins from the fungal species and other microorganisms. Among them, nine proteins were upregulated under nitrogen repletion and five proteins were upregulated under nitrogen limitation, as shown in Table 2. Under nitrogen sufficient condition, the upregulated proteins were involved in cell division and growth (14, 60S ribosomal protein L11, histone H2B, and meiotic expression upregulated protein 14), energy metabolism (ADP/ATP carrier protein-like protein, and adenylate kinase), the glycolytic pathway (fructose-bisphosphate aldolase, and pyruvate decarboxylase), etc. The 60S ribosomal protein L11 and histone H2B take part in nucleic acid replication and cell division. Fructose-bisphosphate aldolase (FBA) catalyzes the reversible reaction between fructose-1, 6-diphosphate, and dihydroxyacetone phosphate (DHAP), plus 3-phosphate glyceraldehyde (G-3-P) [24], which is one of the key enzymes in the glycolytic pathway and affects the rate of glycolysis. It was worth noting that the ADP/ATP carrier protein plays a key role in the maintenance of energetic fluxes in eukaryotic cells, which is an integral protein of the mitochondrial inner membrane that exchanges cytoplasmic ADP for mitochondrial ATP under the conditions of oxidative phosphorylation [25]. All these upregulated proteins revealed that the high level of nitrogen could affect glycolysis and aerobic metabolism, which would promote *A. pullulans* cells to grow faster.

**Table 2:** Identification of differentially expressed protein spots from *A. pullulans* cells under different levels of nitrogen via proteomics analysis with 2D-GE and MALDI-TOF/TOF

Spot no.	Protein name	Putative function	pI	Mascot score	Coverage (%)	Mass (Da)	Fold change	E value
A17	Meiotic expression up regulated protein 14	Cell differentiation	5.44	70	18	39,244	4.93	0.0013
A18	S-adenosylmethionine synthase	Amino acid metabolism	5.75	128	12	43,115	1,000,000^a^	1.9e−009
A46	Pyruvate decarboxylase	Glycolytic pathway	5.57	163	21	64,181	2.97	6e−013
A52	Fructose-bisphosphate aldolase, class II	Carbohydrate metabolism	5.41	282	22	39,637	2.24	7.5e−025
A56	ADP/ATP carrier protein-like protein	Energy metabolism	9.81	64	30	33,892	2.32	0.0045
A57	Adenylate kinase	Energy metabolism	8.84	87	17	30,980	8.36	2.6e−005
A62	ADP/ATP carrier protein-like protein	Energy metabolism	9.81	243	30	33,892	2.14	6e−021
A103	60S ribosomal protein L11	Ribosome	10.08	81	18	21,863	3.10	9.5e−005
A118	Histone H2B	Nucleosome	10.16	159	37	15,047	2.25	1.5e−012
B02	Lysophospholipase Plb2	Lipid metabolism	4.31	44	2	71,790	1,000,000	0.00022
B11	NADH-quinone oxido-reductase	Electron transport	5.71	245	24	82,010	1.68	3.8e−021
B21	Casein kinase II subunit beta	Cell differentiation	4.40	122	7	38,463	2.01	7.5e−009
B24	Nucleosome assembly protein-like protein	Cell differentiation	4.26	299	25	45,383	1.97	1.5e−026
B41	Glyceraldehyde-3-phosphate dehydrogenase	Glycolytic pathway	6.25	260	21	36,436	2.40	1.2e−022

*A17–A118* upregulated proteins under nitrogen repletion; *B02–B41* upregulated proteins under nitrogen limitation

^a^This value means infinite because the protein in the control is not be detected

**Fig. 5:**
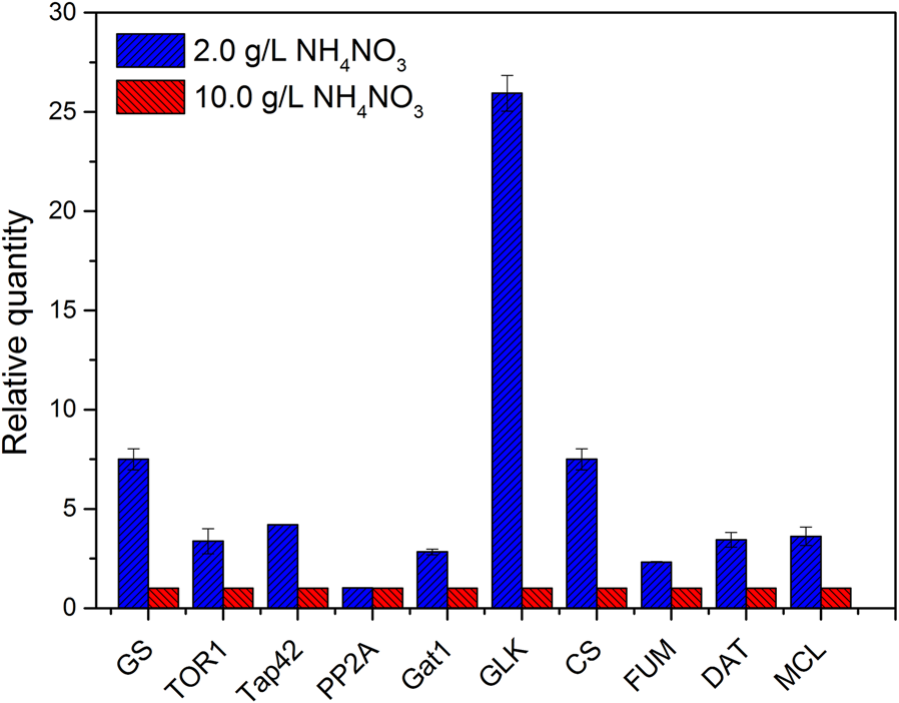
Transcription levels of key genes in the PMA and TOR pathways under the different levels of nitrogen. Data are given as the average of triplicate experiments

**Fig. 6:**
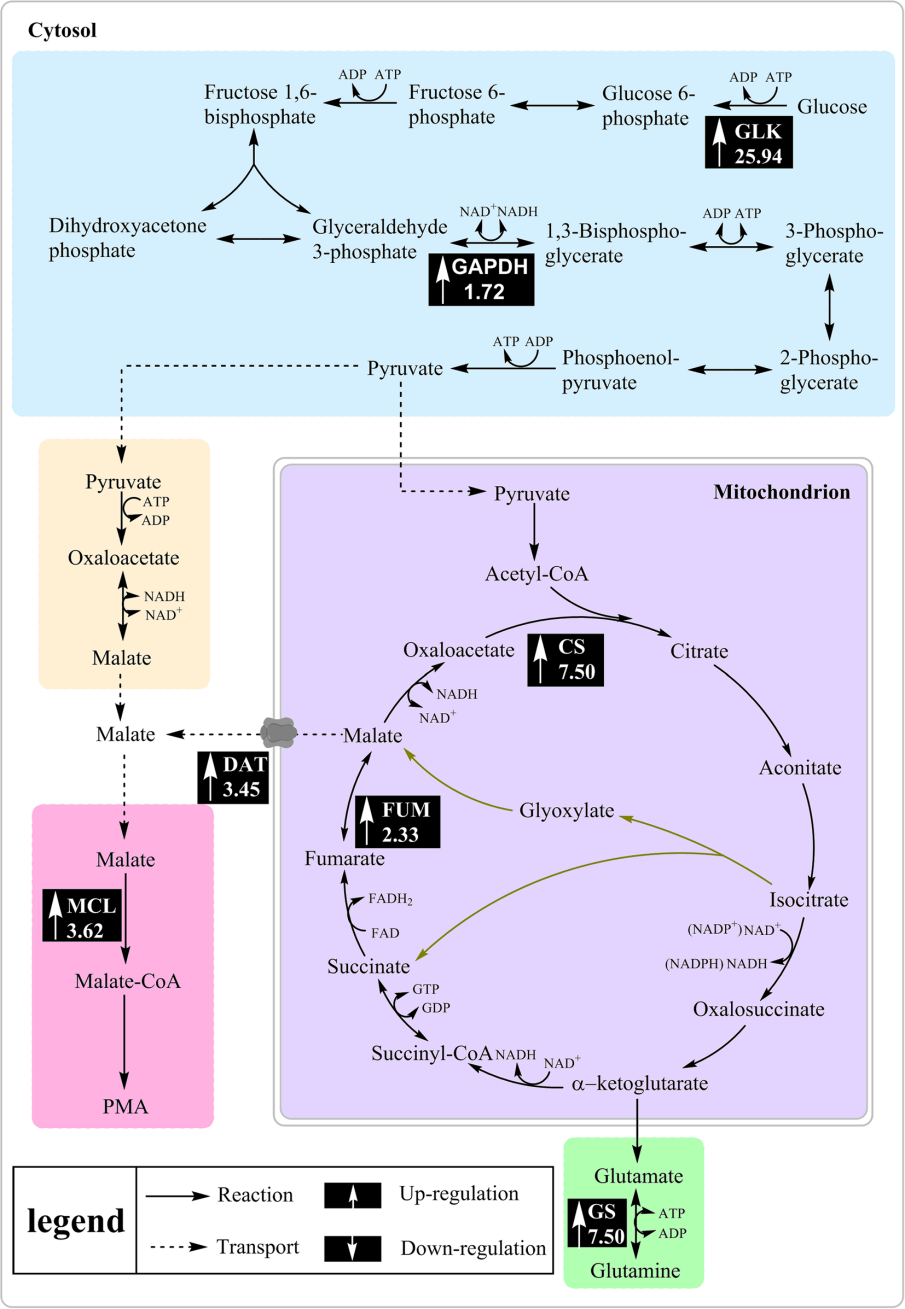
PMA biosynthesis metabolic network and the transcriptional changes of the genes encoding the enzymes catalyzing those steps. *Up arrows* represent the upregulated genes under nitrogen limitation (2 g/L of NH_4_NO_3_)

In comparison, under the nitrogen-limited condition, the upregulated proteins were involved in cell division (casein kinase II subunit beta and the nucleosome assembly protein), the glycolytic pathway (glyceraldehyde-3-phosphate dehydrogenase, GAPDH), and the electron transport chain (NADH-quinone oxidoreductase). GAPDH is an important enzyme that can drive carbon flux to form pyruvate in the glycolytic pathway [26]. Moreover, the expression levels of key genes involved in the PMA biosynthetic pathway were further confirmed by the qRT-PCR method. It is evident in Figs. 5 and 6 that the expression levels of glucokinase (GLK), citrate synthase (CS), and fumarase (FUM) involved in glycolysis and the TCA cycle were upregulated by 25.93, 2.42 and 2.33-fold, respectively, under nitrogen limitation. Mitochondrial dicarboxylate transporter (DAT) was responsible for the transport of malic acid from the mitochondrion to the cytoplasm. In our previous study, the expression level of DAT was upregulated by the inducement of Tween-80, which was beneficial for PMA biosynthesis [14]. In addition, malate-CoA ligase (MCL) was responsible for PMA polymerization from malic acid [27]. As shown in Fig. 5, the expression levels of the genes (*DAT* and *MCL*) were upregulated by 3.09 and 3.25-fold, respectively. These results indicated that carbon flux from glycolysis and the TCA cycle was strengthened to produce more malic acid, and the upregulation of DAT and MCL was responsible for transferring more malic acid from mitochondrion and polymerize them to produce PMA. Furthermore, under nitrogen limitation, the expression levels of the genes (*GS, TOR1, Tap42* and *Gat1*) involved in the TOR signaling pathway were upregulated by 7.49, 3.33, 3.36 and 2.83-fold, respectively. This result further revealed that the TOR pathway, via the Tap42-PP2A branch, was activated and might positively take part in regulating PMA biosynthesis.

### Rapamycin treatment on cell growth and PMA biosynthesis

**Fig. 7:**
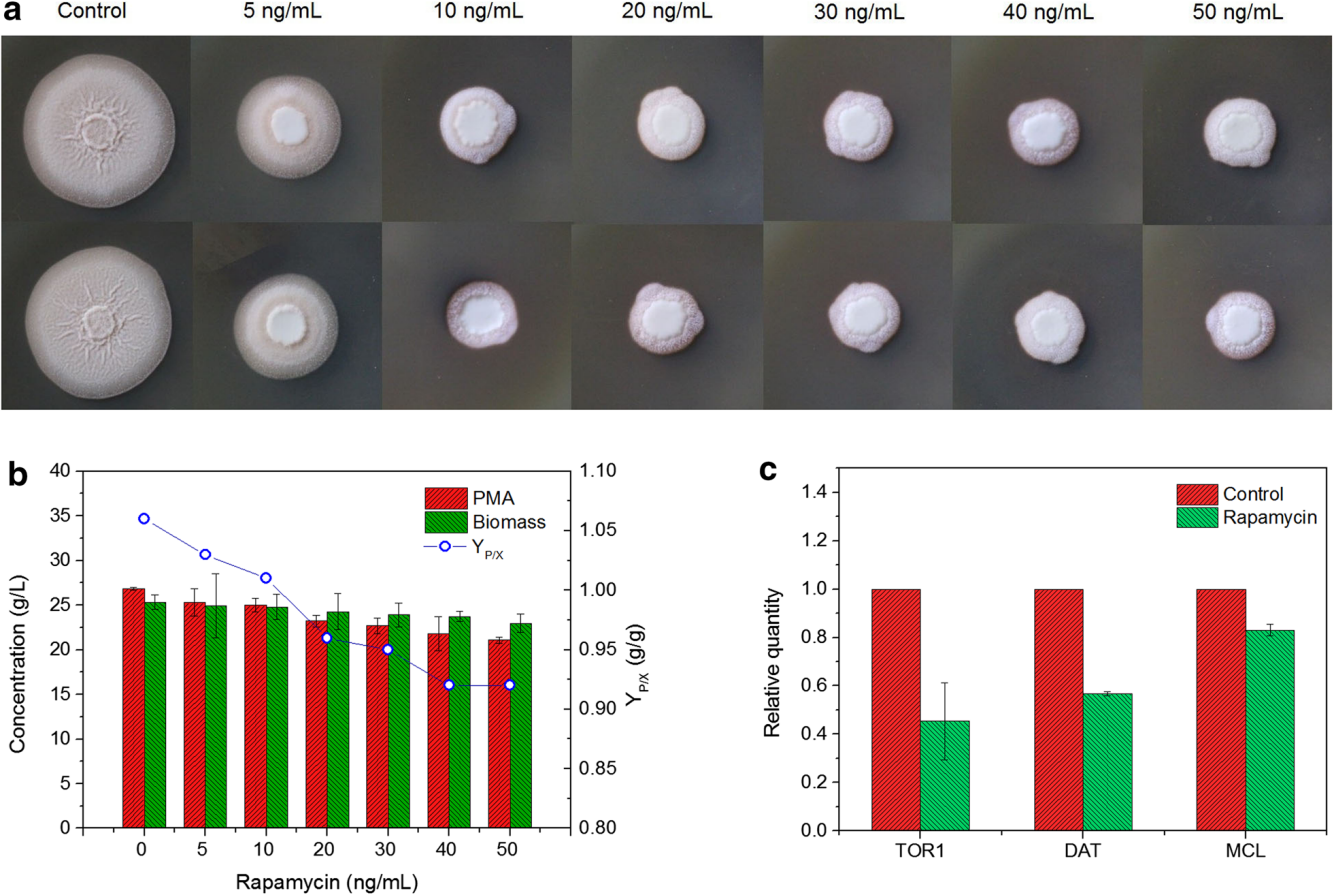
Effect of rapamycin treatment on cell growth and PMA biosynthesis. **a** Growth on PDA plate; **b** Shake flask fermentation; **c** Transcription levels of genes in the PMA and TOR pathways. Yield (Yp/x): the ratio of PMA to cell biomass concentration (g/g). Data are given as the average of triplicate experiments

As previously described, the TOR pathway responds to nutrients and growth factors to orchestrate cell growth and proliferation in eukaryotic cells. Usually, TOR can be inhibited by rapamycin, an immunosuppressant drug. Therefore, we further investigated the role of the TOR signaling pathway on cell growth and PMA biosynthesis under the treatment of rapamycin. As shown in Fig. 7a, radial growth of *A. pullulans* was severely inhibited on a PDA plate when the rapamycin concentration was >5 ng/mL. Correspondingly, the production of PMA in shake flasks decreased gradually (Fig. 7b); after adding 50 ng/mL of rapamycin, the PMA titer was decreased by 21.3 % compared to that in the control. Moreover, the corresponding PMA yield (Yp/x) was also decreased to 0.92 g/g (the control as 1.08 g/g), which showed that PMA biosynthesis was inhibited by rapamycin with a dose-dependent manner. These results suggested that the TOR pathway indeed participated in regulating PMA biosynthesis.

As previously described, the production of PMA under nitrogen repletion was lower than that under nitrogen limitation. The inhibitory effect of rapamycin on PMA biosynthesis was similar to that under nitrogen repletion. The transcription levels of key genes involved in the PMA and TOR pathways after adding 10 ng/mL of rapamycin are shown in Fig. 7c. As the target of rapamycin, the expression level of *TOR1* was obviously downregulated. The transcription levels of *MCL* and *DAT* were downregulated by 0.83 and 0.56-fold, which might be the direct evidence to explain the decrease of the PMA titer.

## Discussion

Culture stress, especially the level of nitrogen, is becoming a useful strategy for the overproduction of organic acids and biopolymers. In this study, the effect of nitrogen availability on PMA biosynthesis was investigated. While a high level of nitrogen favored cell growth, it inhibited the production of PMA. Under nitrogen limitation (2 g/L of NH_4_NO_3_), a final PMA titer of 44.0 ± 3.65 g/L (49.9 ± 4.14 g/L of malic acid after hydrolysis) was achieved at 96 h in a 5-L fermentor, which was increased by 18.3 % compared to that under nitrogen repletion (10 g/L of NH_4_NO_3_). It was noted that, during the whole fermentation process, the ratio of ATP/ADP under nitrogen limitation was also higher than that under nitrogen repletion. According to the biosynthetic pathway of PMA proposed by Willibald et al. [28], adequate energy supply was necessary to polymerize the monomer, L-malic acid, to form PMA. Therefore, more ATP produced under nitrogen limitation was preferable to match the energy demand for the polymerization of PMA.

Transcriptomics analysis resulted in some genes involved in ribosome, ribosome biogenesis, peroxisome, and nitrogen metabolism that were expressed significantly at different levels of nitrogen. After the analysis of proteomics was integrated, it was clear that the levels of nitrogen regulate some proteins involved in cell growth and differentiation, glycolysis and energy metabolism. Under nitrogen repletion, cell growth was obviously faster than that under nitrogen limitation, which was associated with some upregulated proteins such as fructose-bisphosphate aldolase (FBA). Sheng et al. found that (NH_4_)_2_SO_4_ could regulate the fructose-bisphosphate aldolase that led to carbon flux toward the glycolytic pathway for the growth of biomass in *A. pullulans* [29], which was consistent with our experimental data. In comparison, under the nitrogen-limited condition, the upregulated proteins were mainly involved in glycolysis and energy metabolism, which meant producing more ATP and substrates for the synthesis of PMA.

As noted, the reversible oxidative phosphorylation of G-3-P to 1, 3-bisphosphoglycerate (1, 3-BPG) is mediated by GAPDH using NAD^+^ as a cofactor [26]. GAPDH is known as having higher relative activity compared to other enzymes in the glycolytic pathway, and is regarded as a switching control of glycolysis [30]. NADH-quinone oxidoreductase (NDH-1) is the first enzyme in the respiratory chain in mitochondria. It catalyzes oxidation of NADH in the mitochondrial matrix and reduction of quinone in the membrane, and is coupled to proton translocation through the inner mitochondrial membrane [31]. Under nitrogen-limited conditions, the enhanced activity of GAPDH and NDH-1 would strengthen the glycolytic pathway and oxidize more NADH, driving carbon flux toward pyruvate and subsequent malic acid for PMA biosynthesis. In addition, as shown in Figs. 5 and 6, the transcriptional levels of key genes involved in the PMA biosynthetic pathway (e.g., *GLK, FUM, CS, DAT*, and *MCL*) were upregulated under nitrogen limitation. It was worth noting that the expression level of glucokinase (GLK) was upregulated by 25.93-fold, which would extremely promote the rate of glycolysis from glucose.

Moreover, the low level of nitrogen upregulated the transcriptional levels of genes involved in the TOR signaling pathway, as shown in Fig. 5. Glutamine, catalyzed exclusively by glutamine synthetase (GS), is an upstream regulator of the TOR pathway. GS plays an important role not only in providing glutamine, but also as a key regulator in the nitrogen regulatory network in yeast and filamentous fungi [32]. Glutamine starvation affects a subset of TOR-controlled transcription factors including GLN3, RTG1, and RTG3 [33]. Among the downstream effectors of TOR kinase, Tap42-PP2A is the most relevant effector for stress response. Nitrogen starvation and TOR kinase inactivation result in Tap42p dephosphorylation and subsequent dissociation of the Tap42-PP2A and Tap42-PP2A-like phosphatase complex [34, 35], thereby regulating several transcription factors (including Gat1, Gln3, Gaf1, etc.) to drive nitrogen catabolism [36, 37]. Compared to nitrogen repletion, the expression levels of genes (*GS*, *TOR1, Tap42,* and *Gat1*) involved in the TOR pathway were upregulated, indicating that the TOR signaling pathway, via Tap42-PP2A branch, was activated and positively regulated PMA biosynthesis. Furthermore, *A. pullulans* cell growth was obviously inhibited by the rapamycin treatment, accompanied with a dose-depended decrease in the PMA titer. The expression levels of genes (*TOR1, DAT,* and *MCL*) were also downregulated after the treatment of rapamycin. These results further revealed that the TOR pathway indeed participated in regulating cell growth and PMA biosynthesis. In *Fusarium fujikuroi*, Teichert et al. found that TOR kinase is involved in the nitrogen regulation of secondary metabolism [19]. Under nitrogen starvation, protein phosphatase 2A (PP2A)-branch signaling in the downstream TOR pathway can be activated [36].

Among the above upregulated genes, *Gat1* belongs to a conserved family of zinc-finger- containing transcriptional regulators known as GATA-factors, which can activate the transcription of nitrogen catabolite repression (NCR)-sensitive genes when preferred nitrogen sources are absent or limited [38]. It was found that AreA, the ortholog of Gat1, could regulate secondary metabolite biosynthesis in different species. In *F. graminearum*, AreA could regulate the production of the mycotoxins deoxynivalenol (DON) and zearalenone [39]. Disruption of AreA in *Acremonium chrysogenum* could reduce cephalosporin production [40]. In comparison, how it acts on sensitive genes in the PMA biosynthetic pathway via Gat1 or the other TOR-controlled transcription factors were not fully understood.

Under nitrogen limitation, our assumption might be confirmed that more α-ketoglutarate was produced by an activated TCA cycle, which resulted in the production of more GS; then, TOR1 was activated by GS and resulted in the phosphorylation of Tap42. In this case, PP2A was separated from Tap42 and then was inhibited, which will promote the migration of Gat1 into nucleus from the cytoplasm and positively regulate the expression of key genes in the PMA biosynthetic pathway.

## Conclusions

In this study, the level of nitrogen was found to regulate cell growth and PMA biosynthesis. The low level of nitrogen was beneficial for PMA biosynthesis, which could upregulate the expression levels of key genes involved in the PMA biosynthesis pathway. Cell growth and PMA biosynthesis in response to nitrogen might be mediated by the TOR signaling pathway. This study will help us to deeply understand the molecular mechanisms of PMA biosynthesis regulated by the different levels of nitrogen, and to develop an effective process for the production of PMA and malic acid chemicals.

## Additional files

10.1186/s12934-016-0547-y Primers for the gene transcription level analysis.
10.1186/s12934-016-0547-y Totals of differentially expressed genes (DEGs) under nitrogen-sufficient conditions.
10.1186/s12934-016-0547-y Numbers of genes in mainly enriched pathways under nitrogen-sufficient conditions.
