# Supplementary material for: Effects of nitrogen availability on polymalic acid biosynthesis in the yeast-like fungus Aureobasidium pullulans
Source: Microb Cell Fact. 2016 Aug 22;15(1):146. doi: 10.1186/s12934-016-0547-y (PMC4994417; doi:10.1186/s12934-016-0547-y)
Supplement: Supplementary file 1 — 10.1186/s12934-016-0547-y Primers for the gene transcription level analysis. [file 12934_2016_547_MOESM1_ESM.pdf]

Table S1 Primers for the gene transcription level analysis

Genes	Primer name	Primer sequence	Product size (bp)
GS	GS-F	AACTTCGACGGTTCGTCAAC	183
	GS-R	CATAAGGAGAGCAGCGTCGT	
TOR1	TOR1-F	TCATCGCATACAAGCAAAGC	129
	TOR1-R	AGGCACGTACCTTGAGCATT	
Tap42	Tap42-F	CATCCCCGGCTGTGTCA	157
	Tap42-R	AAAGGACGTACAACGCCATGT	
Gat1	Gat1-F	TACTCCTACCACCCAGACCA	141
	Gat1-R	TGCTTCCGCTGACGCTTA	
GLK	GLK-F	GACACTGTCGGAACCCTCAT	169
	GLK-R	TCTCACCAGTGGTCTTGTCG	
FUM	FUM-F	TCTTTGACCATGCTCTGCTG	260
	FUM-R	TAGCCAATGACGGGGTTAAG	
CS	CS-F	TCCCTGGCGAGATTGAGAAG	173
	CS-R	GATAGTCTTGCCACGGAAACG	
DAT	DAT-F	TCTGGACTGGCTTGACTGGAT	155
	DAT-R	ACCAGCGACACCGGAACA	
MCL	Mcl-F	TCTCCCCTCAGTGCATCGA	173
	Mcl-R	CTGTCTGAAGTCGGCGTTGTC	
