# Supplementary material for: Effects of nitrogen availability on polymalic acid biosynthesis in the yeast-like fungus Aureobasidium pullulans
Source: Microb Cell Fact. 2016 Aug 22;15(1):146. doi: 10.1186/s12934-016-0547-y (PMC4994417; doi:10.1186/s12934-016-0547-y)

Figure S1. Total numbers of differentially expressed genes (DEGs) under nitrogen-sufficient conditions.

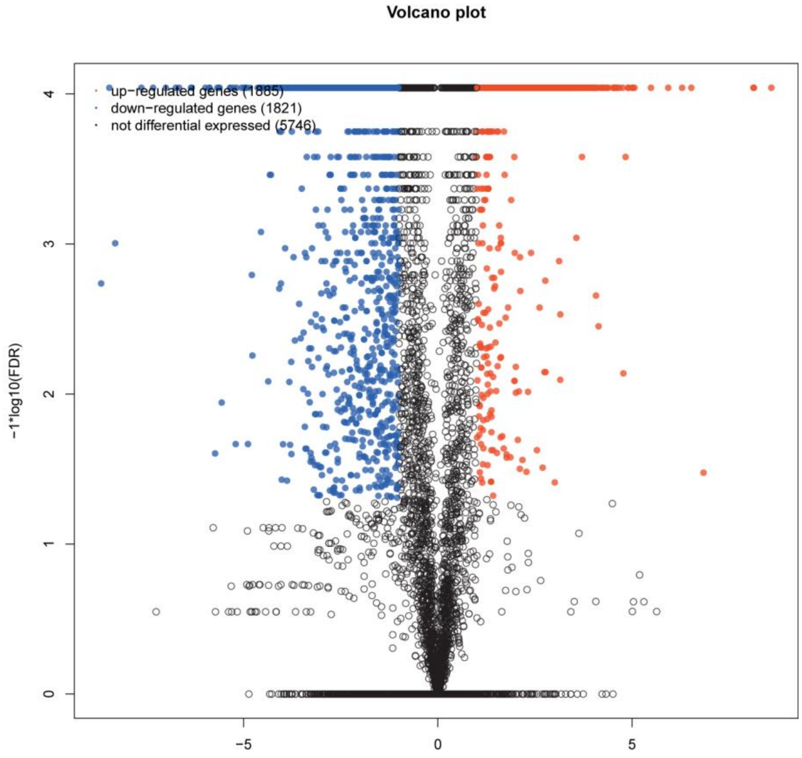

Supplement: Supplementary file 2 — 10.1186/s12934-016-0547-y Totals of differentially expressed genes (DEGs) under nitrogen-sufficient conditions. [file 12934_2016_547_MOESM2_ESM.pdf]
